# Melittin as an Activator of the Autophagy and Unfolded Protein Response Pathways in Colorectal HCT116 Cell Line

**DOI:** 10.61186/ibj.3993

**Published:** 2023-11-21

**Authors:** Mozhdeh Zamani, Farzaneh Bozorg-Ghalati, Pooneh Mokarram

**Affiliations:** 1 Autophagy Research Center, Shiraz University of Medical Sciences, Shiraz, Iran;; 2 Department of Biochemistry, School of Medicine, Shiraz University of Medical Sciences, Shiraz, Iran;; 3 Autophagy Research Center, Department of Biochemistry, School of Medicine, Shiraz University of Medical Sciences, Shiraz, Iran

**Keywords:** Autophagy, Bee venom, Colorectal neoplasms, Unfolded protein response

## Abstract

**Background:**

The potential anticancer effect of melittin has motivated scientists to find its exact molecular mechanism of action. There are few data on the effect of melittin on the UPR and autophagy as two critical pathways involved in tumorigenesis of colorectal and drug resistance. This study aimed to investigate the effect of melittin on these pathways in the CRC HCT116 cells.

**Methods:**

MTT method was carried out to assess the cytotoxicity of melittin on the HCT116 cell line for 24, 48, and 72 h. After selecting the optimal concentrations and treatment times, the gene expression of autophagy flux markers (*LC3-βII* and* P62*) and UPR markers (*CHOP* and *XBP-1s*) were determined using qRT-PCR. The protein level of autophagy initiation marker (Beclin1) was also determined by Western blotting.

**Results:**

MTT assay showed a cytotoxic effect of melittin on the HCT116 cells. The increase in *LC3-βII* and decrease in *P62* mRNA expression levels, along with the elevation in the Beclin1 protein level, indicated the stimulatory role of melittin on the autophagy. Melittin also significantly enhanced the *CHOP* and *XBP-1s* expressions at mRNA level, suggesting the positive role of the melittin on the UPR activation.

**Conclusion:**

This study shows that UPR and autophagy can potentially be considered as two key signaling pathways in tumorigenesis, which can be targeted by the BV melittin in the HCT116 cells. Further in vivo evaluations are recommended to verify the obtained results.

## INTRODUCTION

For a long time, venomous animals such as insects have widely been used in research and medicine^[^^1^^]^. In this regard, it has been shown that various animal toxins have the ability to kill the tumor cells^[^^2^^,^^3^^]^. The BV and its major ingredient melittin have extensively been studied in the past years^[^^4^^]^. Melittin is a small peptide with the chemical formula of C131H228N38O32 and constitutes almost 50% of the dry weight of honeybee venom^[^^5^^]^. It is composed of 26 amino acids with the sequence of GIGAVLKVLTTGLPALISWIKRKRQQ-NH_2_ and molecular weight of 2847.5 Daltons^[^^6^^]^. In addition to powerful hemolytic activity, melittin has the ability to enter the cellular membranes. It can make pores in the phospholipid bilayer membrane for ion transferring, which causes the membrane disruption^[^^6^^]^.

Both BV and melittin have shown strong toxicity in various types of tumor cells^[^^7^^-^^12^^]^. Melittin is an interesting candidate for cancer therapy since cancer cells are highly sensitive to membrane-disrupting compounds^[^^2^^]^. Therefore, the combination therapy, including BV products and conventional chemo-therapeutic agents, may lead to synergistic anticancer effects and reduce the doses required for each of them^[^^13^^]^. For instance, ovarian cancer cells and Hodgkin's lymphoma cells better responded to the combination therapy of melittin and cisplatin compared to the treatment with each of them alone^[^^14^^,^^15^^]^.

Disturbance in the synthesis, folding, and modification of the proteins results in ER stress in the cells, which activates the UPR signaling pathway^[^^16^^,^^17^^]^. The UPR activation helps cells mange this stress by decreasing the misfolded or unfolded proteins in the ER^[^^17^^-^^19^^]^. Autophagy signaling is another pathway, which has a key role in keeping the cellular ER homeostasis in the normal condition. The autophagy-related genes are highly upregulated during the ER stress to protect the cell survival through lipid homeostasis, mitochondrial metabolism, and recycling of degraded materials such as proteins^[^^20^^,^^21^^]^. During this process, dysfunctional organelles and damaged proteins are engulfed in vesicles called autophagosomes. Finally, fusion of these autophagosomes with lysosomes leads to the degradation and recycle of their contents^[^^20^^]^. Autophagy and UPR pathways promote the cancer cell growth and survival through adaptation to the stressful conditions such as hypoxia and nutrient starvation, however; these pathways cause cancer cell death. Therefore, they are known as dual swords in cancer due to these opposing functions, which make them suitable potential targets for cancer treatment^[^^22^^,^^23^^]^. 

The effect of the BV and its main constituent melittin on the autophagy and UPR pathways have been reported in different cancers such as osteosarcoma^[^^24^^]^, glioblastoma^[^^25^^]^, lung^[^^26^^]^, liver^[^^27^^]^, and cervix cancers^[^^28^^]^. In spite of various investigations on the role of melittin in the growth inhibition and apoptosis induction in colon cancer cells^[^^12^^,^^29^^]^, to the best of our knowledge, there is no report about the function of melittin in autophagy and UPR pathways in the mentioned cells. Considering the high prevalence of CRC worldwide, the significant mortality caused by this disease^[^^30^^]^, and the importance of autophagy and UPR pathways in cancer and drug resistance^[^^19^^,^^31^^]^, the present study was aimed to investigate the effect of melittin on these pathways in HCT116 CRC cell line.

## MATERIALS AND METHODS

**Cell culture**

The HCT116 cells (Pasteur Institute of Iran, Tehran) were grown in an incubator (37 °C, CO_2_ 5%) and cultured in RPMI 1640 Gluta MAX™ medium (Biowest, Nuaillé, France) supplemented with fetal bovine serum (10%; Gibco™, EU-Approved, South American) and 1% penicillin-streptomycin (Biowest). 

**MTT assay**

MTT method was used to investigate the antiproliferative activity of melittin (Sigma Aldrich, St. Louis, USA) in the HCT116 cell line in vitro, following a previously described protocol^[^^29^^]^. Briefly, after seeding the 2 × 10^3^ of HCT116 cells in each well of 96-well plate and reaching a proper confluency (80%), the cells were treated with varying concentrations of melittin, ranging from 0.25 to 16 µg/ml for 24, 48, and 72 hours. Then after adding MTT reagent (Sigma Aldrich; 5 mg/ml, 20 μM), the cells were incubated for another 4 hours, and the absorbance was measured at 570 and 630 nm using an ELISA reader (Mikura Ltd. Pocklington, England). Eventually, according to the cell viability curves, the IC_50_ values were determined.

**Gene expression analysis**

qRT-PCR was utilized to assess the melittin effect on autophagy and UPR-related genes. After treatment with melittin at 1 and 2 µg/ml concentrations for 24 h, total RNA extraction of the cells was conducted by the RNX-Plus RNA extraction kit from CinnaGen (Iran), based on the instruction provided by the manufacturer. The concentration and purity of the extracted RNA were determined through the optical density measurement at 260/280 nm by applying a NanoDrop pectrophotometer (Thermo Fisher Scientific, Waltham, MA, USA). The gel electrophoresis was applied under denaturing conditions to evaluate the RNA integrity. The complementary DNA synthesis was carried out by a cDNA Synthesis Kit from Thermo Fisher Scientific. Gene amplification using RealQ PCR 2× Master Mix SYBR Green low ROX^®^ (Amplicon, Stenhuggervej, Denmark) and specific primers (Methabion, Germany), as described in Table 1, using the ABI real-time PCR 7500 system (Applied Biosystems Inc., Foster City, CA, USA). The analysis of the relative expression changes for the desired genes was conducted using the ΔΔCt method.

**Western blotting**

Following a 24-hour exposure to 1 and 2 μg/ml of melittin, cells were collected and lysed by NP-40 lysis buffer alongside phosphatase and protease inhibitors (Sigma Aldrich) at an ice-cold temperature. Next, the protein bands were separated using SDS-PAGE (15%), followed by transferring onto a nitrocellulose membrane. To avoid the nonspecific connection of antibodies, the membrane was incubated with 5% non-fat milk (Sigma Aldrich) at a cold room temperature for two hours. GAPDH mouse monoclonal antibody (0411, Santa Cruz Biotechnology, Texas, USA) and Beclin1 rabbit monoclonal antibody (D40C5, Cell Signaling Technology, Massachusetts, USA) were added at a 1:1000 ratio to the membrane and left at 4 °C overnight. After three-time washing with Tris-buffered saline containing 0.1% Tween^®^ 20 detergent, secondary antibodies, including antimouse IgG (1:10000, Sigma Aldrich, A8924, St. Louis, Missouri, USA) and HRP-conjugated goat antirabbit (1:5000, AP7181, Razi Biotech, Iran) were added to detect GAPDH and Beclin 1 protein bands, respectively. The membrane and antibodies were then incubated at ambient temperature for two hours. The chemiluminescence signals were determined using a ChemiDoc MP Imaging system (Bio-Rad, USA). The blots intensity was measured using densitometry software Image Lab. Finally, based on the control level of GAPDH protein, all bands were normalized.

**Table 1:** The primer sequences of the UPR and autophagy markers

**Genes**	**Forward primers (5'-3')**	**Reverse primers (5'-3')**
*CHOP*	GCTCTGATTGACCGAATGG	TTCTGGGAAAGGTGGGTAG
*XBP-1s*	TGCTGAGTCCGCAGCAGGTG	GCTGGCAGGCTCTGGGGAAG
*LC3-βII*	AACGGGCTGTGTGAGAAAAC	AGTGAGGACTTTGGGTGTGG
*P62*	AATCAGCTTCTGGTCCATCG	TTCTTTTCCCTCCGTGCTC
*GAPDH*	CGACCACTTTGTCAAGCTCA	AGGGGTCTACATGGCAACTG

**Statistical analysis**

All the data was analyzed using GraphPad Prism 8 software (GraphPad Prism, RRID: SCR_002798). One-way analysis of variance (ANOVA) and Tukey's post-hoc tests were used for data analysis. Data are illustrated as mean ± standard deviation of three different replicates (n = 3). The *p* values less than 0.05 were statistically significant. 

## RESULTS

**Melittin cytotoxicity in the HCT116 cell line**

**Fig. 1:**
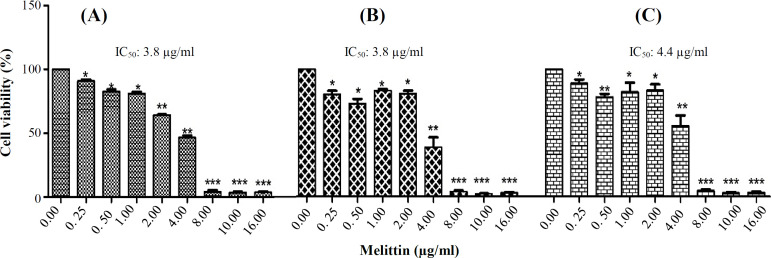
Effect of melittin on the growth of HCT116 colon cancer cell line. The cells were exposed to the different concentrations of melittin for 24 h (A), 48 h (B), and 72 h (C). The cell viability was assessed by MTT assay. Data are reported as mean ± SD of three independent assays (n = 3; ^*^p < 0.05; ^**^p < 0.01; ^***^p < 0. 001 compared with control (untreated HCT116) cells

The melittin cytotoxicity on the HCT116 cells was assessed by MTT method after treatment with melittin at concentrations of 0, 0.25, 0.5, 1, 2, 4, 8, 10, and 16 µg/ml. The MTT findings revealed a significant cytotoxicity for melittin on HCT116 cells (Fig. 1). The viability of melittin-treated HCT116 cells (0.25 to 16 μg/ml) was significantly reduced compared to untreated cells at 24, 48, and 72 h. According to the MTT results and the obtained IC_50_ value for melittin, 1 and 2 μg/ml were chosen as optimal melittin concentrations, and time of 24 h was selected as exposure time for the next procedures in HCT116 cells.

**Impact of melittin on the autophagy in HCT116 cells**

**Fig. 2:**
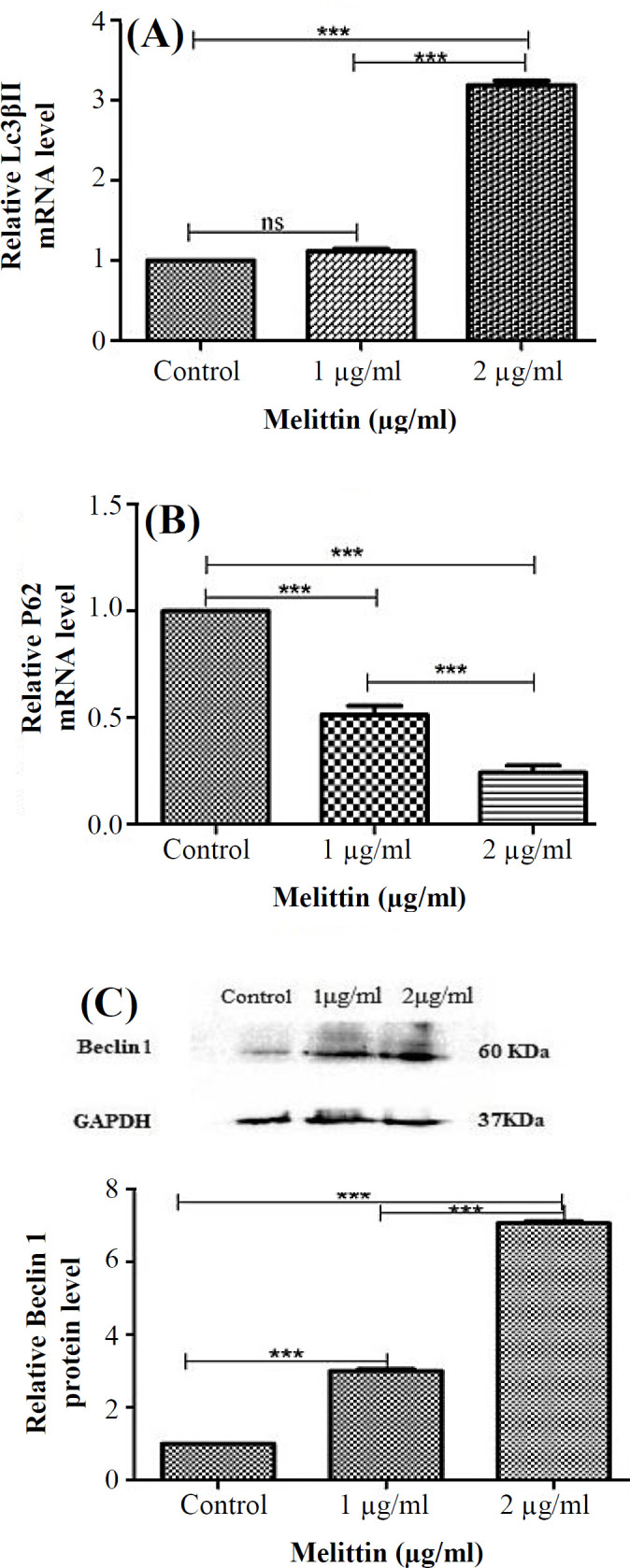
Induction of the gene expression of *LC3-βII* and *P62 *and protein expression of Beclin1 by melittin. The relative mRNA expression levels are presented for *LC3-βII* (A) and *P62* (B), and protein expression level is presented for Beclin1 (c) in HCT116 cells for 24 h. Data are reported as mean ± SD of three independent assays (n = 3; ^***^p < 0.001)

The melittin-mediated effect on autophagy was studied by subjecting HCT116 cells to 1 and 2 µg/ml of melittin at 24 h. The autophagy flux biomarkers at mRNA levels, including *LC3-βII* and *P62*, and protein level of autophagy initiation biomarker, Beclin1, were evaluated using RT-PCR and Western blotting, respectively. As illustrated in Figure 2, treatment of HCT116 cells with 2 µg/ml of melittin significantly enhanced the mRNA expression of *LC3-βII* compared to the control after 24 h (3.18-fold;* p* < 0.001; Fig. 2A). 

Besides, the *P62* expression level decreased significantly at 1 and 2 µg/ml of melittin after 24 h (0.49- and 0.20-folds, respectively;* p* < 0.001; Fig. 2B). The real-time PCR amplification and melting curve plots of *LC3-βII*, *P62*, and *GAPDH* genes are shown in **Figures S1-S3**, respectively. The Western blotting results indicated a remarkable increase in the protein level of Beclin1 in HCT116 cells after melittin treatment (1 and 2 µg/ml) for 24 h (3- and 7.06-folds, respectively;* p* < 0.001; Fig 2C).

**Melittin effect on the UPR pathway in HCT116 cells**

**Fig. 3:**
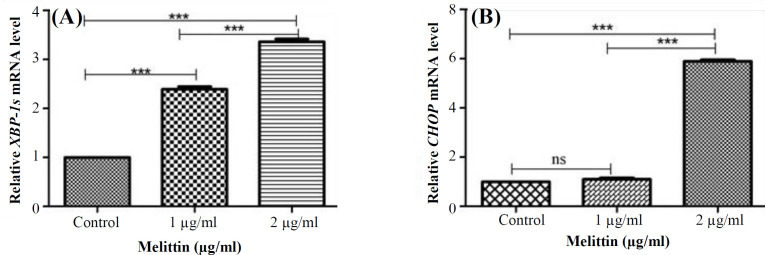
Increased expression levels of *CHOP* and *XBP-1s* genes by melittin. The relative mRNA expression levels are presented for *XBP-1s* (A) and *CHOP* (b) in HCT116 cells for 24 h. Data are reported as mean ± SD of three independent assays (n = 3; ^***^p < 0.001 compared with control)

To investigate the melittin effect on the UPR pathway, we used qRT-PCR to measure the mRNA levels of the two genes associated with UPR; *XBP-1s* as the downstream marker of the IRE1 arm and *CHOP* as the downstream marker of PERK arm. The expression of *XBP-1s *mRNA was significantly enhanced in melittin-treated HCT116 cells compared to the control untreated cells at 24 h (2.39- and 3.36-folds for 1 and 2 μg/ml, respectively; *p* < 0.001; Fig. 3A). Melittin also significantly enhanced the *CHOP *mRNA expression level at 2 µg/ml concentration after 24 h (5. 99-folds;* p* < 0.001; Fig. 3b,). The real-time PCR amplification and melting curve plots of *CHOP* and *XBP-1s* genes are shown in **Figures S4 and S5**, respectively.

## DISCUSSION

The increasing prevalence of cancer has been evident in recent years^[^^32^^]^. Although recommended treatment strategies such as surgery, radio- and chemotherapy contribute to the management of this disease, the mortality rate is still growing. Therefore, deep scientific studies are needed to discover new treatment strategies^[^^33^^]^. In spite of modern medical progress, drugs derived from plants and animals are still well-known owing to their ability in the prevention and treatment of various diseases, including cancer^[^^2^^]^. Hence, this study was designed to better understand the mechanism of action of the main BV component, melittin, as an animal-derived compound, on two cancer-related pathways, autophagy and UPR, in HCT116 cells. 

Our experimental study described a cytotoxicity for melittin on the human HCT116 cell line. The 1 and 2 µg/ml concentrations of melittin and time of 24 h were selected for further evaluating both autophagy and UPR pathways in the cells. Our findings revealed that melittin increased the UPR biomarkers, *CHOP* and *XBP-1s*, 24 h after treatment. Increase in the Beclin1 and *LC3-βII* expression levels and decrease in *P62* mRNA expression level demonstrated the activation of autophagy by melittin after 24 h. 

The present study is the first description of how melittin affects autophagy and UPR pathways in the CRC HCT116 cell line. According to the evidence available, the main proposed mechanisms by which melittin exerts its anticancer activity include cell cycle arrest induction, cancer cell growth suppression, and induction of apoptosis and necrosis in various cancer cells^[^^2^^]^. In this context, it has been shown that BV induces apoptosis and inhibits the growth of colon cancer cells through the suppression of NF-κB and activation of DR4 and DR5^[^^29^^]^. Additionally, melittin induces apoptosis in ovarian cancer cells by elevating the expression of DR3, DR4, and DR6 death receptors and inhibiting the JAK2/STAT3 signaling pathway^[^^14^^]^. Melittin can also induce Bcl2-dependent and caspase-3-dependent apoptosis in U937 leukemia cells by inhibiting the AKT signaling pathway^[^^34^^]^. Jeong et al. indicated that melittin inhibited the cell motility and invasion of breast cancer cells by preventing the PI3K/Akt/mTOR cascade, which is the upstream regulator of autophagy^[^^35^^]^. Melittin also prevents the HepG2 liver cancer cell progression through the PTEN upregulation mediated by HDAC2 and suppression of the PI3K/Akt pathways^[^^36^^]^. Tipgomut and colleagues also exhibited that melittin inhibited cellular proliferation and apoptosis induction through the Bcl-2 upregulation and MADD downregulation in Chago-K1 human bronchogenic carcinoma cells^[^^37^^]^.

Despite the importance of understanding the impact of melittin on different cellular pathways, there is currently a limited number of investigations exploring its effect on the UPR and the autophagy pathways^[^^24^^-^^28^^]^. UPR and autophagy are two key pathways during ER stress^[^^18^^]^. The UPR pathway activates autophagy; in contrast, autophagy limits the UPR pathway by reducing the ER stress^[^^38^^]^. The prolonged and severe stress of the ER that is not eliminated by the UPR and autophagy pathways triggers apoptosis in order to remove the damaged cells^[^^38^^,^^39^^]^. Therefore, to maintain cellular homeostasis, the crosstalk of UPR, autophagy, and apoptosis pathways determines the cellular fate during the ER stresses^[^^40^^]^. According to the emerging evidence, disruption of autophagy, UPR, and apoptosis plays an important role in colorectal tumorigenesis and drug resistance^[^^16^^,^^41^^]^. In this regard, Lv et al. showed that melittin could inhibit the growth of liver tumors by inducing the mitochondrial-dependent apoptosis pathway^[^^27^^]^. However, it also activates the autophagy pathway in hepatocellular carcinoma cells, which has a protective role against apoptosis and contributes to drug resistance. Based on this study, autophagy inhibitors such as chloroquine increase the toxic effect of melittin in liver cancer cells^[^^27^^]^. In another study conducted in 2021, Wang and colleagues demonstrated that UM-6, a novel anticancer fusion peptide based on melittin, is capable of activating both autophagy and apoptosis pathways in uterine cervix cancer cells and inhibiting their growth^[^^28^^]^. In 2022, Yu et al. reported that autophagy activation by BV induced apoptosis (autophagy-mediated apoptosis) in lung cancer cells through the mTOR pathway^[^^26^^]^. Similarly, Song et al., found that using autophagy inhibitors such as hydroxychloroquine decreased bee venom-induced apoptosis^[^^40^^]^. Also, Fan et al. described that the melittin gene-transfected human osteosarcoma cell line (MG63) triggered UPR-mediated apoptosis through a significant increase in the XBP1 and CHOP proteins level^[^^24^^]^. Bazi and colleagues also reported BV as an activator of the UPR pathway and UPR-related apoptosis in the A172 glioblastoma cell line^[^^25^^]^. While some investigations have reported that melittin restricts the proliferation and promotes apoptosis in colon cancer cells^[^^12^^,^^29^^]^, the autophagy and UPR pathways have not been targeted so far in these cells. In the same line with the studies mentioned earlier on the activation role of BV and melittin on autophagy and UPR pathways in cancer cells^[^^24^^-^^28^^]^, similar results obtained in this study in the HCT116 cell line 24 hour after treatment with melittin. 

In contrast to previous studies, herein, we evaluated both autophagy and UPR pathways, simultaneously, in HCT116 cells after melittin treatment. Since this research is the first report on the effect of melittin on these pathways in CRC cells, there are some limitations, including a lack of normal cell line and also an appropriate apoptosis assay. To precisely recognize the mechanism of action of melittin in CRC, we recommend overcoming these limitations along with in vivo studies to confirm the obtained results.

## CONCLUSION

Melittin exhibited anticancer activity against the CRC HCT116 cell line. Based on the findings from this study, it appears that the anticancer mechanism of melittin in the HCT116 cell line may be related to the UPR- and autophagy-mediated cell death. 

## DECLARATIONS

### Acknowledgments

The authors would also like to express their sincere gratitude to Dr. Nasrin Shokrpour at the Research Consultation Center (RCC) of Shiraz University of Medical Sciences (Shiraz, Iran) for her valuable contribution in English editing. No artificial intelligence-assisted technologies have been used in this study.

### Ethical approval

This study was approved by the Ethics Committee of Shiraz University of Medical Sciences, Shiraz, Iran (ethical code: IR.SUMS.REC.1401.127).

### Consent to participate

Not applicable.

### Consent for publication

All authors reviewed the results and approved the final version of the manuscript.

### Authors’ contributions

MZ: coordinated and designed the study, acquired funding, and wrote the manuscript; FBG: conducted the experiments; PM: coordinated and designed the study; MZ, FBG, and PM analyzed and interpreted the data. 

### Data availability

The raw data supporting the conclusions of this article are available from the authors upon reasonable request.

### Competing interests

The authors declare that they have no competing interests. 

### Funding

This work was financially supported by Shiraz University of Medical Sciences, Shiraz, Iran (grant no.25013)

### Supplementary information

The online version contains supplementary materials. 
